# Muscle Mitochondrial Oxidative Capacity and Cognitive Decline Over up to a Decade: Sex Differences

**DOI:** 10.1111/acel.70637

**Published:** 2026-07-21

**Authors:** Qu Tian, Anjay Ambegaonkar, Luigi Ferrucci

**Affiliations:** ^1^ Translational Gerontology Branch, Intramural Research Program National Institute on Aging, NIH Baltimore Maryland USA

## Abstract

Higher skeletal muscle oxidative capacity is associated with higher cognitive function and preserved brain structure. The relationship between mitochondria and brain health suggests sex differences, but whether the relationship with cognition shows sex differences and underlying mechanisms is unknown. We analyzed the relationships between mitochondrial oxidative capacity and up to 12‐year prospective cognitive data in 506 older participants (mean age = 74.4 years, 58% Women) and examined sex differences, using linear mixed‐effects models adjusted for demographics. Cognitive composite scores were computed using multiple cognitive measures. Muscle oxidative capacity was assessed as the post‐exercise recovery rate of phosphocreatine (kPCr) via phosphorus‐31 MR spectroscopy. We tested mediation effects of blood‐based clinical markers. Cross‐sectionally, higher kPCr was associated with higher cognitive scores (*p* < 0.001 overall, *p* = 0.001 men, *p* = 0.029 women), and there was no significant kPCr‐by‐sex interaction (*p* = 0.308). Longitudinally, there was a significant kPCr‐by‐sex interaction (*p* = 0.042). The association of higher kPCr with slower cognitive decline was prominent in men (*p* = 0.077), particularly executive function (*p* = 0.010) and processing speed (*p* = 0.049). In men, fasting glucose and hemoglobin A1C mediated both cross‐sectional and longitudinal associations between kPCr and cognition (all *p* < 0.05, 23.1% by glucose). Total protein and globulin also mediated the cross‐sectional association in men to a lesser extent (9.5%–9.9%). In women, ESR, hemoglobin, and hematocrit mediated the cross‐sectional association (all *p* < 0.05, 22.7% by hematocrit). Muscle mitochondria are linked to cognition and predict cognitive decline primarily in men. Underlying mechanisms appear to differ by sex, likely through a metabolic‐drive pathway in men and a hematologic‐inflammatory pathway in women. Omics studies are warranted to elucidate the sex‐specific biological processes.

## Graphical abstract

Skeletal muscle mitochondrial oxidative capacity is linked to cognition and predicts cognitive decline primarily in men. Underlying mechanisms appear to differ by sex, likely through a metabolic‐drive pathway in men and a hematologic‐inflammatory pathway in women.

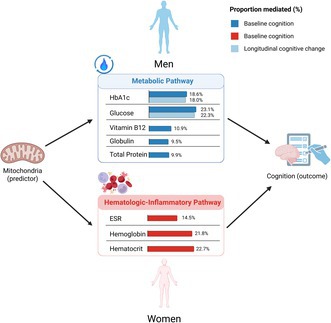

## 1 Introduction

Through electron microscopy in the 1950s, mitochondria were first identified as the powerhouse of cells with their double‐membraned structure and cristae in the inner membrane as a key site for energy production. Mitochondrial dysfunction is recognized as a leading hallmark of aging, primarily driven by reduced ATP production, increased reactive oxygen species due to impaired mitophagy, somatic mutation, and deletions of mitochondrial DNA, with most evidence supported in vitro (Lopez‐Otin et al. 2023). In humans, in vivo evidence supporting the relationship between mitochondria and aging phenotypes and function is emerging. Baseline mitochondrial oxidative capacity assessed in skeletal muscle via non‐invasive MR spectroscopy is not only associated with cognitive performance but also predicts brain atrophy, mobility decline, and risks of cognitive impairment and dementia (Tian, Lee, et al. 2024; Tian, Bilgel, et al. 2023; Tian, Mitchell, et al. 2022; Tian, Greig, et al. 2024). It remains unclear whether mitochondrial health is associated with longitudinal changes in cognitive function over time. Potential mechanisms underlying mitochondria and cognition may include neural stem cell depletion, impaired neurogenesis, and neuroinflammation, as supported predominantly in animal models (Khacho et al. 2019; Kaplan et al. 2015). In addition, sex differences are suggested in the relationship between mitochondrial health and the brain, with some evidence from blood‐based markers. Mitochondrial DNA copy number in the blood is associated with cognition and brain iron deposition, more prominent in men than in women (Casanova et al. 2024; Tian, Zweibaum, et al. 2024; Zhang et al. 2023), but the underlying mechanisms are unclear. Data from blood‐based lipid markers reveal sex‐specific associations with brain aging, which suggest the important role of mitochondria in sex‐specific mechanisms (Tian, Mitchell, et al. 2023).

In this study, we aimed to extend prior research by (1) examining longitudinal associations between muscle mitochondrial oxidative capacity and cognitive change over time, (2) testing whether these associations would be different in men compared to women, and (3) investigating mediating effects of a large panel of blood‐based clinical markers.

## 2 Methods

### 2.1 Study Population

**FIGURE 1:**
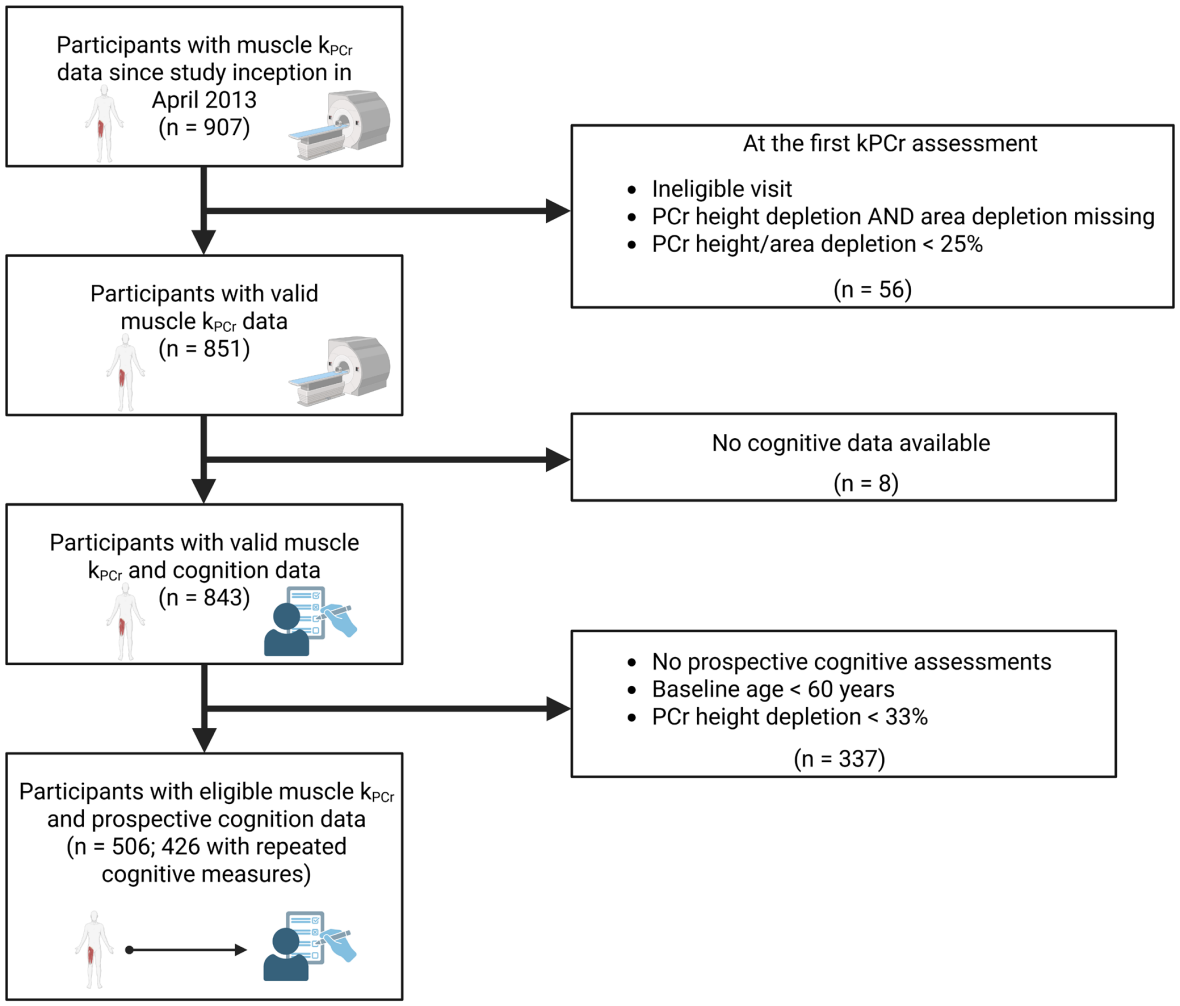
Flow chart of the sample selection. Ineligible visits included cognitive assessments administered at home. Created in BioRender. Ambegaonkar, A. (2026) https://BioRender.com/us2n29o.

Participants were selected from the Baltimore Longitudinal Study of Aging (BLSA), a longitudinal study of human aging with continuous enrollment since 1958 (Shock et al. 1984; Ferrucci 2008). Participants were cognitively unimpaired and without chronic disease or serious illness at the study enrollment. Once enrolled, participants were followed up over time. Follow‐up intervals depend on age (every 4 years for those aged 60 and younger, every 2 years for those aged 60–79, and annually for those aged 80 and older). The assessment of skeletal muscle oxidative capacity via MR spectroscopy began in April 2013. We chose the first assessment of muscle mitochondrial oxidative capacity as the independent variable (i.e., predictor) in this study. We analyzed a sample of 506 participants aged 60 and older with data on the first assessment of skeletal muscle oxidative capacity and concurrent and/or prospective longitudinal data on cognitive measures between 2013 and 2025 (Figure 1). We chose to analyze data in participants older than 60 years of age who undergo more prominent biological and cognitive changes than young to middle‐aged adults. The first assessment of skeletal muscle oxidative capacity was considered as the index visit. Of 506, 426 (84%) had repeated cognitive measures spanning up to a decade during an average follow‐up of 6.1 years. Of 506 participants, 471 (93%) are scheduled to return or have returned to the study for follow‐up. Only 35 (7%) did not return to the study due to withdrawal (*n* = 13), disqualification (*n* = 2), termination (*n* = 1) and death (*n* = 19). The Institutional Review Board of the National Institutes of Health approved the BLSA protocol. All BLSA participants provided written informed consent at each visit.

### 2.2 Skeletal Muscle Oxidative Capacity (Independent Variable/Predictor)

In vivo phosphorus‐containing metabolites were collected from the quadriceps muscles of the left thigh via ^31^P‐MR Spectroscopy at a 3T Achieva MR scanner (Philips, Best, The Netherlands). Detailed data acquisition and pre‐processing steps were published previously (Tian, Lee, et al. 2024). In brief, to deplete phosphocreatine (PCr) in the quadriceps muscles, participants were instructed to perform a fast and intense ballistic knee extension exercise while positioned supine in the bore of the scanner with a 10‐cm, flat surface coil (PulseTeq, Surrey, United Kingdom) secured over the vastus lateralis muscle of the left thigh. A total of 75 ^31^P‐MRS spectra with 6‐s temporal resolution for each spectrum were obtained with a pulse‐acquire sequence 60 s before, approximately 30 s during, and 360 s after exercise. The length of the exercise phase was monitored to achieve between 33% and 66% reduction in PCr peak height and not to exceed 42 s. Spectra were processed using jMRUI (version 5.2). Maximum muscle oxidative capacity was quantified as the recovery rate of PCr after exercise, denoted as kPCr. kPCr was determined by fitting time‐dependent changes in PCr peak area using the following mono‐exponential function:

$$\mathrm{PCr}\left(t\right)=\mathrm{PC}\,r0+\Delta \,\mathrm{PCr}\times \left(1-e-t/\tau \right)$$

where PCr_0_ was the PCr signal amplitude at the end of the exercise and the beginning of the recovery, ΔPCr was the decrease in PCr observed from baseline to the end of the exercise, τ was the PCr recovery time constant, and k_PCr_ was the PCr recovery rate constant determined as 1/τ. The PCr recovery rate provides an indirect in vivo measure of muscle oxidative capacity and has been validated against ex vivo mitochondrial respiration (Gonzalez‐Freire et al. 2018). Higher kPCr indicates higher oxidative capacity. In this study, kPCr data with at least 33% height depletion was included for analysis. An optimal level of PCr depletion, suggested as 33%–66%, is necessary to obtain reliable kPCr because extreme levels of PCr depletion can lead to low pH which alters the recovery dynamics (Meyerspeer et al. 2020).

### 2.3 Cognition and Diagnoses of Cognitive Impairment and Dementia

Various cognitive functions were measured using a comprehensive neuropsychological battery, including global mental status, verbal memory, verbal fluency, attention, processing speed, executive function, manual dexterity, and visuospatial ability (Tian, An, et al. 2023). Global mental status was assessed by the Mini‐Mental State Examination (MMSE). Verbal memory was measured using the immediate recall of the California Verbal Learning Test (CVLT). Verbal fluency was assessed by letter fluency and category fluency tests. Attention was assessed by the Trail Making Test A (TMT‐A). Executive function was assessed by the Delta Trail Making Test (Delta TMT), defined as the difference between the times required to complete the Trail Making Test B (TMT‐B) and TMT‐A. Processing speed was assessed by the digit symbol substitution test (DSST). Manual dexterity was assessed using the Purdue Pegboard Test. Visuospatial ability was assessed using the Card Rotation Test. We computed cognitive composite scores by averaging the standardized *Z* scores of each cognitive measure. Signs of *Z* scores for TMT‐A and Delta TMT were flipped before computation, so their directionality was consistent with other cognitive scores, with higher values indicating better cognitive performance. To examine prospective change over time, we focused on cognitive data concurrent at and after the first assessment of skeletal muscle oxidative capacity during clinical visits, from April 2013 to December 2025. To check the robustness of the findings, we repeated the analyses by restricting to data points without cognitive impairment or dementia. Adjudication of cognitive impairment and dementia follows a standard procedure in the BLSA (Tian, Bilgel, et al. 2022). Mild cognitive impairment was diagnosed using the Petersen criteria (Petersen et al. 1997). Alzheimer's Disease and dementia were diagnosed using the National Institute of Neurological and Communicative Disorders and Stroke‐Alzheimer's Disease and Related Disorders Association, and the Diagnostic and Statistical Manual, the third edition, revised, respectively (American Psychiatric Association 1987).

### 2.4 Clinical Markers of Interest as Mediators

We tested a large panel of blood‐based clinical markers measured at the first assessment of skeletal muscle mitochondrial oxidative capacity. Blood samples were collected from the antecubital vein between 7 and 8 AM. Participants fasted overnight and did not smoke, engage in physical activity, or take medications before sample collection. We focused on clinical markers with less than 10% of missing values. For each participant, missing values were imputed using the nearest available data relative to the index visit. A total of 64 blood markers, spanning from glycemic control, lipid, metabolic, inflammatory, and physiological domains, were included in this study and tested as potential mediators (Table S1).

### 2.5 Statistical Analysis

We used linear mixed‐effects (LME) models to examine associations between the first assessment of kPCr and prospective longitudinal trajectories of cognitive performance. The first assessment of kPCr was considered as the index visit (i.e., time 0). Data for visits occurring after the index visit were modeled with positive time intervals (+1 years, +2 years, etc.). Measures of kPCr (predictor) and cognitive outcomes were standardized to *Z* scores based on mean and SD at the index visit. Models were adjusted for age at the index visit, sex, education, and PCr depletion. Fixed effects included covariates and their respective interactions with the time interval. Random effects included participant‐specific intercepts and slopes. In cases where models failed to converge, random effects were reduced to random intercepts only. Cross‐sectional associations were reported for the index visit, and prospective longitudinal associations were reported for subsequent visits up to 12 years. We focused on the cognitive composite scores as the primary outcome and explored individual cognitive measures in separate LME models.

To formally test sex differences in the association between kPCr and cognition, we added 2‐way (kPCr:sex) and 3‐way (kPCr:sex:interval) interaction terms to the LME for cross‐sectional and longitudinal effects. Sex‐specific estimates were then extracted from interaction models using the *emmeans* package in R. We repeated analyses in those who were cognitively normal by removing data points at and after symptom onset of cognitive impairment. We also repeated the analyses by excluding outlying observations of cognitive composite scores more than 5 SD. To assess whether potential survival bias would affect results, we performed sensitivity analysis by excluding those 35 participants who lost follow‐up. Because of a possible ceiling effect of MMSE, we also created a composite score excluding MMSE and analyzed the longitudinal association with kPCr as a sensitivity analysis. We additionally adjusted for physical activity, apolipoprotein E ε4 carrier status, vascular conditions, and race as sensitivity analyses. All LME models were fit using the *nlme* package in RStudio version 4.5.1 (Boston, MA). In this exploratory analysis with the primary focus on cognitive composite scores, statistical significance was set at two‐sided *p* < 0.05.

We further tested the mediation effects of clinical markers on sex‐specific associations between muscle mitochondrial oxidative capacity and cognition using the ‘mediation’ package in R. We set muscle mitochondrial oxidative capacity (kPCr) as the predictor/exposure and each clinical marker measured concurrently at the index visit as individual mediators. We examined two cognitive outcomes, including cognitive composite scores at the index visit and changes in cognitive composite scores over time. Changes in cognitive composite scores were computed as individual‐specific slopes using LME. Mediation models were adjusted for the same set of covariates in LME. We used 500 simulations per model in the quasi‐Bayesian resampling simulations. In the mediation analysis, we reported (1) the direct effect of kPCr (exposure) on cognitive outcomes while controlling for the clinical marker, (2) the indirect effect which was the product of regression coefficient of kPCr (predictor) on the clinical marker and the regression coefficient of the clinical marker on cognitive outcomes, (3) total effect which was the sum of direct and indirect effects, and (4) the proportion mediated. In this exploratory mediation analysis, we set significance at two‐sided *p* < 0.05. We also corrected multiple testing using the Benjamine–Hochberg method. In addition, we compared these clinical markers between men and women using independent *t*‐tests.

## 3 Results

Participants' demographics and characteristics at the index visit are presented in Table 1. The mean age of the study sample was 74.4 (SD = 8.2) years. 58% were women. 26% were Black. There were no significant differences in age, education, apolipoprotein E ɛ4 carrier status, kPCr, or vascular conditions between men and women (Table 1). Men were more likely to be physically active (Table 1). Of 506, 426 (84%) had repeated cognitive measures during an average follow‐up of 6.1 years.

**TABLE 1:** Participants' characteristics at the time of mitochondrial oxidative capacity assessment.

	Total (*n* = 506)	Men (*n* = 212)	Women (*n* = 294)	*p*
	Mean ± SD or *N* (%)			
Demographics				
Age, years	74.4 ± 8.2	75.2 ± 8.3	73.9 ± 8.2	0.073
Black	131 (26%)	39 (18%)	92 (31%)	0.002
Education				0.283
< High school	1 (0.2%)	1 (0.5%)	0 (0%)	
High school	20 (4.0%)	5 (2.4%)	15 (5.1%)	
Some college	61 (12%)	23 (11%)	38 (13%)	
College graduates	98 (19%)	39 (18%)	59 (20%)	
Post college	326 (64%)	144 (68%)	182 (62%)	
Physical activity	(*n* = 499)	(*n* = 209)	(*n* = 290)	0.003
Inactive	50 (10%)	18 (8.7%)	32 (11%)	
Low	199 (40%)	74 (35%)	125 (43%)	
Moderate	148 (30%)	58 (28%)	90 (31%)	
Very active	102 (20%)	59 (28%)	43 (15%)	
Apolipoprotein E ε4 carriers	123 (25%) (*n* = 483)	47 (24%) (*n* = 199)	76 (27%) (*n* = 284)	0.500
Vascular conditions	254 (50%)	115 (54%)	139 (47%)	0.145
kPCr, s^−1^	0.0205 ± 0.0051	0.0209 ± 0.0053	0.0203 ± 0.0049	0.172
Cognition				
Mini‐Mental State Exam	28.5 ± 1.5	28.2 ± 1.5	28.6 ± 1.5	< 0.001
CVLT immediate recall	51.3 ± 12.2	47.4 ± 12.3	54.1 ± 11.4	< 0.001
Category fluency	15.8 ± 3.7	14.8 ± 3.6	16.5 ± 3.7	< 0.001
Letter fluency	14.5 ± 4.2	14.2 ± 4.2	14.8 ± 4.1	0.082
TMT part A (sec)	32.8 ± 12.9	34.5 ± 15.4	31.5 ± 10.6	0.014
Delta TMT (sec)	50.7 ± 33.9	53.7 ± 32.8	48.7 ± 34.5	0.107
Card rotation test	83.3 ± 38.2	89.6 ± 39.8	78.6 ± 36.4	0.002
Digit symbol substitution test	41.6 ± 10.9	38.1 ± 10.6	44.2 ± 10.4	< 0.001
Pegboard dominant hand	12.3 ± 2.0	11.6 ± 2.1	12.8 ± 1.8	< 0.001
Pegboard non‐dominant hand	12.0 ± 2.0	11.6 ± 2.0	12.2 ± 1.9	< 0.001

*Note: p*‐values are based on independent *t*‐tests for continuous variables or chi‐squared tests for categorical variables as appropriate. Vascular conditions indicate the presence of the following: myocardial infarction, congestive heart failure, angina or chest pain due to transient ischemic attack, coronary artery disease, stroke, peripheral artery disease, hypertension, and varicose veins.

Abbreviations: CVLT, California Verbal Learning Test; TMT, Trail Making Test.

### 3.1 Overall Associations Between Muscle Mitochondrial Function and Cognitive Outcomes

In the overall sample, cross‐sectionally, higher kPCr was associated with higher cognitive composite score (β ± SE: 0.0897 ± 0.0231, *p* < 0.001), and select cognitive measures, including TMT‐A (β ± SE: 0.0757 ± 0.0285, *p* = 0.008), card rotation test (β ± SE: 0.1450 ± 0.0448, *p* = 0.001), letter fluency (β ± SE: 0.1421 ± 0.0432, *p* = 0.001), category fluency (β ± SE: 0.1052 ± 0.0407, *p* = 0.010), DSST (β ± SE: 0.0958 ± 0.0406, *p* = 0.019), and pegboard dominant hand (β ± SE: 0.0748 ± 0.0329, *p* = 0.024) (Table 2). Longitudinally, in the overall sample, kPCr was not associated with changes in any cognitive outcomes (Table 2).

**TABLE 2:** Associations between mitochondrial oxidative capacity and cognitive function and interaction effects with sex.

Cross‐sectional	Overall sample (*n* = 506)		Sex‐specific	
	Model 1: sex‐adjusted	Model 2: kPCr‐sex interaction	Men (*n* = 212)	Women (*n* = 294)
	β (SE), *p*	β (SE), *p*	β (SE), *p*	β (SE), *p*
Composite score (all included)	0.0897 (0.0231), < 0.001	0.0459 (0.0450), 0.308	0.1143 (0.0332), 0.001	0.0684 (0.0313), 0.029
Composite score (MMSE excluded)	0.0910 (0.0233), < 0.001	0.0324 (0.0453), 0.474	0.1085 (0.0335), 0.001	0.0761 (0.0315), 0.016
MMSE	0.0700 (0.0422), 0.097	0.1477 (0.0816), 0.071	0.1486 (0.0598), 0.013	0.0009 (0.0572), 0.987
CVLT immediate	0.0462 (0.0400), 0.248	0.1397 (0.0777), 0.073	0.1209 (0.0575), 0.036	−0.0188 (0.0539), 0.728
TMT‐A	0.0757 (0.0285), 0.008	0.0527 (0.0557), 0.344	0.1037 (0.0410), 0.012	0.0510 (0.0388), 0.189
Delta TMT	0.0686 (0.0417), 0.101	−0.0285 (0.0813), 0.726	0.0559 (0.0599), 0.351	0.0844 (0.0564), 0.136
Card Rotation	0.1450 (0.0448), 0.001	0.0616 (0.0871), 0.480	0.1782 (0.0643), 0.006	0.1166 (0.0607), 0.055
Letter fluency	0.1421 (0.0432), 0.001	0.0064 (0.0841), 0.940	0.1458 (0.0622), 0.019	0.1395 (0.0585), 0.018
Category fluency	0.1052 (0.0407), 0.010	0.0308 (0.0793), 0.698	0.1219 (0.0586), 0.038	0.0911 (0.0551), 0.099
DSST	0.0958 (0.0406), 0.019	0.0172 (0.0790), 0.828	0.1051 (0.0581), 0.071	0.0879 (0.0553), 0.112
Pegboard dominant hand	0.0748 (0.0329), 0.024	0.0305 (0.0642), 0.635	0.0908 (0.0474), 0.056	0.0603 (0.0446), 0.177
Pegboard non‐dominant hand	0.0148 (0.0358), 0.680	−0.0607 (0.0697), 0.384	−0.0169 (0.0515), 0.742	0.0438 (0.0486), 0.368

Longitudinal	Model 1: Sex‐adjusted	Model 2: kPCr‐sex‐interval interaction	Men (*n* = 212)	Women (*n* = 294)
	β (SE), *p*	β (SE), *p*	β (SE), *p*	β (SE), *p*
Composite score (all included)	0.0012 (0.0027), 0.670	0.0110 (0.0054), 0.042	0.0070 (0.0040), 0.077	−0.0040 (0.0037), 0.284
Composite score (MMSE excluded)	0.0015 (0.0029), 0.611	0.0111 (0.0056), 0.048	0.0074 (0.0041), 0.074	−0.0037 (0.0039), 0.333
MMSE	−0.0025 (0.0053), 0.632	0.0136 (0.0104), 0.191	0.0042 (0.0074), 0.572	−0.0094 (0.0074), 0.203
CVLT immediate	−0.0036 (0.0051), 0.484	0.0009 (0.0101), 0.931	−0.0031 (0.0074), 0.678	−0.0039 (0.0070), 0.570
TMT‐A	0.0070 (0.0104), 0.505	0.0210 (0.0206), 0.308	0.0183 (0.0152), 0.230	−0.0027 (0.0141), 0.847
Delta TMT	0.0027 (0.0054), 0.617	0.0343 (0.0108), 0.001	0.0198 (0.0076), 0.010	−0.0145 (0.0076), 0.057
Card rotation	0.0011 (0.0035), 0.743	0.0128 (0.0068), 0.062	0.0077 (0.0049), 0.117	−0.0051 (0.0048), 0.290
Letter fluency	0.0046 (0.0039), 0.230	0.0096 (0.0076), 0.208	0.0097 (0.0055), 0.082	0.0000 (0.0053), 0.993
Category fluency	−0.0030 (0.0042), 0.472	0.0057 (0.0083), 0.493	−0.0000 (0.0060), 0.998	−0.0057 (0.0058), 0.321
DSST	0.0040 (0.0037), 0.282	0.0125 (0.0074), 0.090	0.0106 (0.0054), 0.049	−0.0019 (0.0051), 0.707
Pegboard dominant hand	−0.0063 (0.0044), 0.150	−0.0043 (0.0087), 0.624	−0.0085 (0.0063), 0.177	−0.0043 (0.0061), 0.483
Pegboard non‐dominant hand	0.0047 (0.0043), 0.282	0.0130 (0.0085), 0.129	0.0114 (0.0062), 0.065	−0.0016 (0.0060), 0.790

*Note:* Beta values for TMT‐A and Delta TMT were sign‐flipped to be consistent with the directionality of other cognitive measures; higher value indicates better cognitive performance. All models were adjusted for age at the time of kPCr assessment, education, and PCr depletion. Sex‐specific estimates were extracted from interaction models (model 2).

Abbreviations: CVLT, California Verbal Learning Test; DSST, digit symbol substitution test; MMSE, Mini‐Mental State Exam; TMT, Trail Making Test.

### 3.2 Mitochondria‐By‐Sex Interaction Effects and Sex‐Specific Associations

**FIGURE 2:**
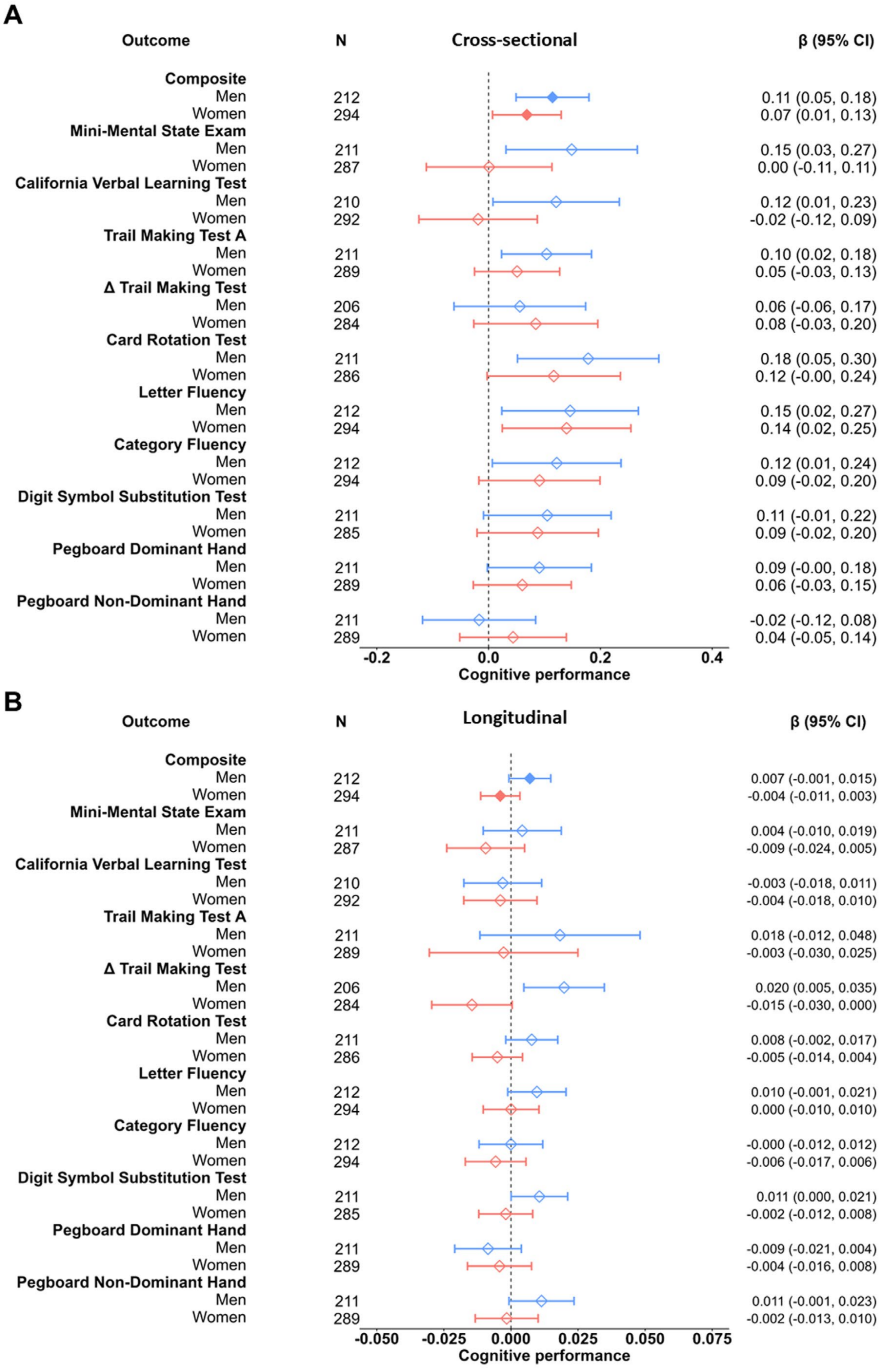
Sex‐specific associations between kPCr and cognitive outcomes. Panel A: Cross‐sectional associations between skeletal muscle mitochondrial oxidative capacity and cognition at baseline; Panel B: Longitudinal associations between skeletal muscle mitochondrial oxidative capacity and subsequent changes in cognition.

Cross‐sectionally, kPCr‐by‐sex interactions showed trends towards significance in associations with MMSE (*p* = 0.071) and CVLT immediate recall (*p* = 0.073) (Table 2, Figure 2A), indicating potential sex differences. Specifically, higher kPCr was associated with greater cognitive performance on MMSE (β ± SE: 0.1486 ± 0.0598, *p* = 0.013) and CVLT (β ± SE: 0.1209 ± 0.0575, *p* = 0.036) in men, not in women. kPCr‐by‐sex interactions were not statistically significant in associations with other cognitive measures, but notably, sex‐specific associations showed more prominent associations in men than in women (Table 2, Figure 2A). In men, higher kPCr was associated with higher cognitive performance in TMT‐A (β ± SE: 0.1037 ± 0.0410, *p* = 0.012), card rotation test (β ± SE: 0.1782 ± 0.0643, *p* = 0.006), letter fluency (β ± SE: 0.1458 ± 0.0622, *p* = 0.019), category fluency (β ± SE = 0.1219 ± 0.0586, *p* = 0.038), and the composite score (β ± SE: 0.1143 ± 0.0332, *p* = 0.001) (Table 2) (Figure 2A). In women, higher kPCr was associated with greater cognitive performance on letter fluency (β ± SE: 0.1395 ± 0.0585, *p* = 0.018) and the composite score (β ± SE: 0.0684 ± 0.0313, *p* = 0.029) (Table 2, Figure 2A).

Longitudinally, kPCr‐sex‐interval three‐way interactions were significant in associations with changes in cognitive composite scores (*p* = 0.042) and delta TMT (*p* = 0.001) and showed trends towards significance in associations with changes in card rotation test (*p* = 0.062) and DSST (*p* = 0.09), indicating sex differences. These longitudinal associations were significant or showed trends towards significance in men, and not in women. In men, higher kPCr was associated with or showed trends towards slower decline in cognitive composite score (β ± SE: 0.0070 ± 0.0040, *p* = 0.077), delta TMT (β ± SE: 0.0198 ± 0.0076, *p* = 0.010) and DSST (β ± SE: 0.0106 ± 0.0054, *p* = 0.049) (Table 2) (Figure 2B). In women, kPCr was not associated with changes in any cognitive outcomes (Table 2) (Figure 2B).

After removing cognitive data points at and after the symptom onset of cognitive impairment, the kPCr‐sex‐interval interaction effects remained similar in associations with changes in delta TMT (*p* = 0.001) and composite scores (*p* = 0.085) (Table S2a). The associations with changes in delta TMT (*p* = 0.031) and DSST (*p* = 0.058) remained prominent in men (Table S2a). The kPCr‐sex‐interval interaction effect on the association with changes in cognitive composite scores remained significant after the removal of 2 outlying composite scores (*p* = 0.042). Results remained similar in those participants who remained on the study for follow‐up (β [SE], *p*‐value for cross‐sectional sex‐adjusted association: 0.0860 (0.0234), < 0.001; kPCr‐by‐sex interaction: 0.0375 (0.0457), 0.411; Longitudinal sex‐adjusted association: 0.0012 (0.0028), 0.671; kPCr‐sex‐interval interaction: 0.0110 (0.0054), 0.044). Associations between kPCr and cognitive composite score excluding MMSE remained similar (Table 2). Additional adjustment for physical activity, apolipoprotein E ε4 carrier status, vascular conditions, and race did not substantially alter the observed associations and kPCr‐by‐sex interaction effect (Table S2b).

### 3.3 Mediation Effects of Clinical Markers on Muscle Mitochondria and Cognitive Composite Scores

Among 64 blood‐based clinical markers, a group of markers significantly mediated the association between kPCr and cognitive composite scores, with distinct patterns in men and women (Figure 1, Table S3a–d).

**FIGURE 3:**
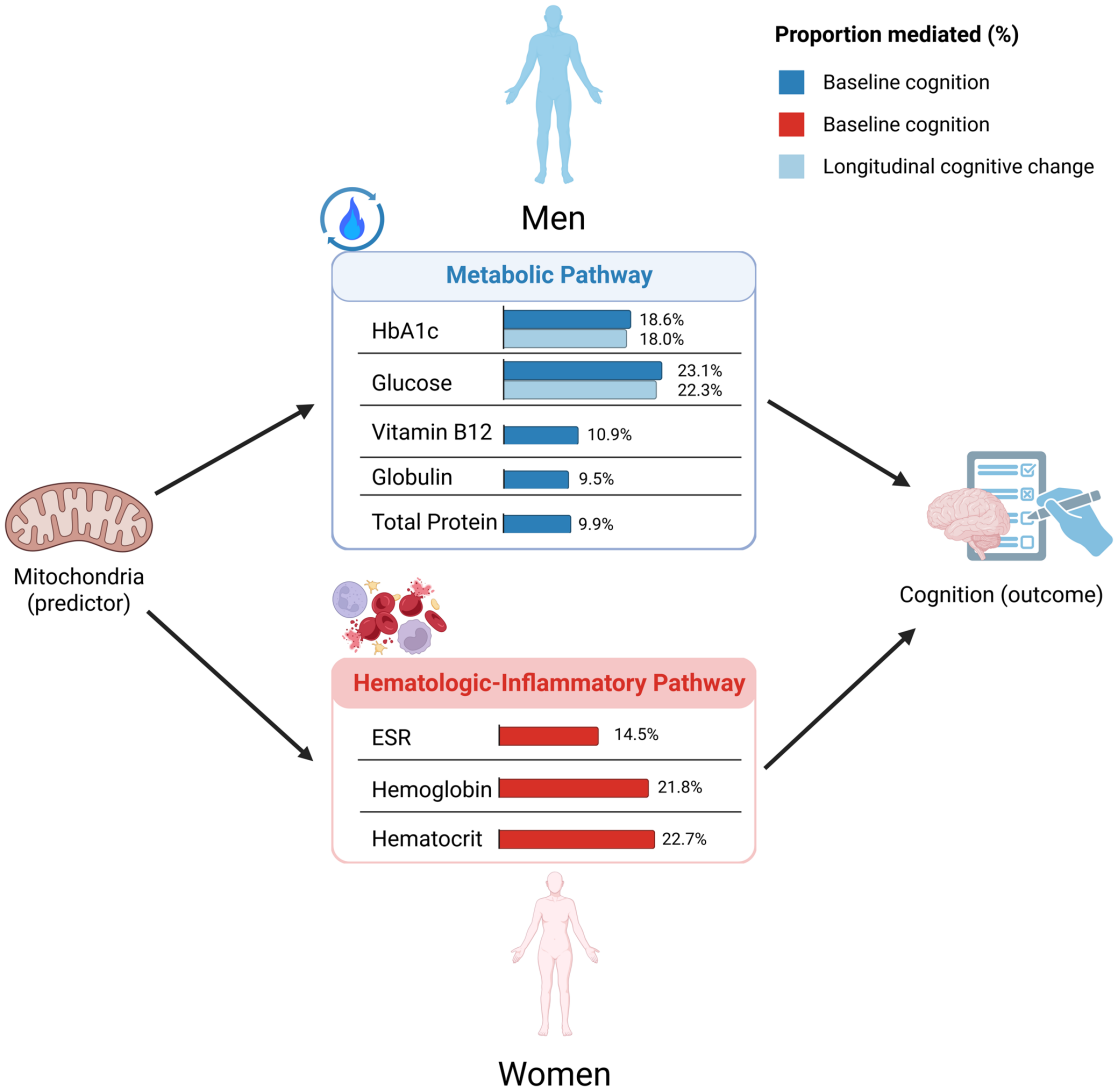
Significant mediation effects of clinical markers on the associations between muscle mitochondrial oxidative capacity and cognitive composite scores in men and women. Created in BioRender. Ambegaonkar, A. (2026) https://BioRender.com/m3zqd5v.

In men, hemoglobin A1c (HbA1c), glucose, vitamin B12, globulin, and total protein mediated the cross‐sectional associations of kPCr with cognitive composite scores (mediation effect *p*‐value, proportion mediated: *p* = 0.024, 18.6%; *p* = 0.004, 23.1%; *p* = 0.040, 10.9%; *p* = 0.048, 9.5%; and *p* = 0.028, 9.9%, respectively; Table S3a, Figure 3). HbA1c and glucose also mediated the associations with longitudinal changes in cognitive composite scores (mediation effect *p*‐value, proportion mediated: *p* = 0.012, 18.0%; and *p* = 0.024, 22.3%, respectively; Table S3b, Figure 3). Compared with women, men had higher levels of glucose and HbA1c and lower levels of vitamin B12, globulin, and total protein (all *p* < 0.05 except HbA1c: *p* = 0.058) (Table S1). No other clinical markers showed significant mediation effects (Table S3a,b).

In women, erythrocyte sedimentation rate (ESR), hemoglobin, and hematocrit mediated the cross‐sectional association with cognitive composite scores (mediation effect *p*‐value, proportion mediated: *p* = 0.032, 14.5%; *p* = 0.012, 21.8%; and *p* = 0.004, 22.7%, respectively; Table S3c, Figure 3). Compared with men, women had higher ESR and lower hemoglobin and hematocrit levels (all *p* < 0.05) (Table S1). No other clinical markers showed significant mediation effects (Table S3c,d). These mediation effects at two‐sided *p* < 0.05 did not survive multiple testing corrections (Table S3a–d).

## 4 Discussion

Higher in vivo muscle mitochondrial oxidative capacity is associated with preserved cognitive function and slower rates of decline over time, and the longitudinal association is prominent in men. Mediation analyses of blood‐based biomarkers reveal different mediating pathways between men and women. Metabolic markers, indicated by fasting glucose and hemoglobin A1c, may explain muscle mitochondrial health and cognition in men, while inflammatory and hematologic markers seem to underlie this relationship in women.

The positive cross‐sectional association between muscle oxidative capacity and cognition is consistent with previous reports that used either blood‐ or muscle‐based mitochondrial measures (Tian, Lee, et al. 2024; Zhang et al. 2023; Tian et al. 2025). Mitochondrial health appears to specifically connect to cognitive measures involving motor planning and speed components (Tian, Lee, et al. 2024; Xia et al. 2025; Krtichevsky et al. 2021; Rosano et al. 2024). Recent data suggest that fat infiltration and inflammation may play a role in the relationship between muscle mitochondria and cognition (Tian, Lee, et al. 2024). We extended prior research by investigating whether muscle mitochondria are connected to subsequent cognitive change over time. We presented novel findings that higher muscle mitochondrial oxidative capacity may be associated with less cognitive decline, but this longitudinal association was primarily found in men, not women.

Our sex‐specific analyses revealed consistent sex differences in associations of muscle mitochondrial oxidative capacity with baseline cognition and cognitive decline over time. Cross‐sectionally, the association between mitochondrial oxidative capacity with either cognitive composite score or individual cognitive score appeared more prominent in men than in women, although the sex‐by‐mitochondria interaction terms were not statistically significant. The stronger association in men is in line with previous cross‐sectional reports using either blood‐based mitochondrial DNA copy number or muscle bioenergetics (Zhang et al. 2023; Krtichevsky et al. 2021). Longitudinally, significant the sex‐by‐mitochondria interaction terms on composite score and delta TMT suggested clear sex differences in the association of muscle mitochondria with cognitive decline over time. Muscle mitochondrial may have more predictive value in subsequent cognitive change, particularly executive function, in men than in women.

Sex differences in the relationship between mitochondria and brain health are understudied, but emerging evidence indicates that such differences may be present. For instance, mitochondrial DNA copy number measured in the blood is related to iron deposition in the brain, localized in the basal ganglia, and this relationship is primary in men, not in women (Casanova et al. 2024; Tian, Zweibaum, et al. 2024). The lack of association between mtDNAcn and brain iron in women may be due to blood loss from lifetime menstruation (Coad and Pedley 2014). Other studies examining lipid metabolites in relation to the progression of brain aging reveal different lipid profiles between men and women; lower beta‐oxidation rate and short‐chain acylcarnitines in women, and higher long‐chain ceramides and very long‐chain triglycerides in men are associated with accelerated brain aging (Tian, Bilgel, et al. 2023). Findings suggest that mitochondrial health plays a role in brain aging but mechanisms may differ by sex and where lipid metabolism occurs.

To understand potential mechanisms, we expanded prior research by testing the mediation effects of a large panel of blood‐based clinical biomarkers on the mitochondria‐cognition relationship. We found different clinical markers that mediated the relationship between mitochondrial oxidative capacity and cognition between men and women. Metabolic‐related markers, including glucose and hemoglobin A1c, were found to mediate associations in men, including associations with both baseline cognition and cognitive decline over time, with vitamin B12 additionally mediating the cross‐sectional association. In men, liver health, indicated by total protein and globulin, also mediated the muscle mitochondria‐cognition relationship to a smaller magnitude than metabolic‐related markers. By contrast, hematologic‐inflammatory markers, including ESR, hemoglobin, and hematocrit, were found to mediate the cross‐sectional association in women. These novel findings suggest sex‐specific biological processes underlying the role of muscle mitochondrial health in cognition, which are also supported by prior evidence. Men and women have shown different plasma lipid biomarkers of accelerated brain aging, with lower beta‐oxidation rate and short‐chain acylcarnitines in women and higher long‐chain ceramides and very long‐chain triglycerides in men. These sex‐specific lipid profiles may suggest different locations where lipid metabolism occurs (Tian, Bilgel, et al. 2023). In the present study, women had lower hematocrit and hemoglobin than men, which are related to reduced beta‐oxidation rate and mitochondrial oxidative phosphorylation through reduced oxygen delivery, which is in line with previous findings that reduced beta‐oxidation rate is associated with accelerated brain aging in women, not in men (Tian, Mitchell, et al. 2023). In women, low hemoglobin and hematocrit are also related to low iron, likely due to blood loss from the menstrual cycle, which is in line with previous findings that the association between mitochondrial DNA copy number and brain iron is prominent in men, not in women (Coad and Pedley 2014). Impaired perfusion or hypoxia may contribute to the relationship between muscle mitochondria and cognition more in women than in men. By contrast, men had higher glucose and HbA1c than women. The insulin signaling may be strongly interfered with long‐chain ceramides and very long‐chain triglycerides, which are associated with accelerated brain aging in men, not in women. Taken together, these may suggest that mitochondrial health is linked to cognition, perhaps through a metabolic, energetic pathway in men and a hematologic‐inflammatory pathway in women. These sex‐specific pathways may serve as potential clinical targets for interventions aimed at slowing cognitive decline. Studies involving omics markers are warranted to further elucidate the mechanisms underlying these sex differences.

This study has notable strengths. First, the examination of both composite and individual cognitive scores allowed us to not only identify the overall relationship between mitochondrial health and cognition but also determine the specificity of cognitive domains. Second, repeated measures of cognitive data up to a decade of follow‐up allowed us to investigate the predictive role of mitochondria in future cognitive decline. Third, the investigation of a panel of blood‐based biomarkers revealed potential sex‐specific mechanisms underlying the relationship between mitochondrial health and cognition. Fourth, the additional sensitivity analyses during the cognitive normal period suggest that results are robust and not substantially affected by cognitive impairment. This study is not without limitations. First, the study population is relatively healthier and has more educational attainment than the general older population, so findings from this study may have been underestimated. Second, the study sample is modest which reduces statistical power especially in multiple testing corrections for mediation effects. Future studies with larger samples and longer follow‐ups are needed to confirm these findings.

## 5 Conclusions

In conclusion, higher skeletal muscle mitochondrial oxidative capacity is consistently associated with cognitive function in both men and women and linked to cognitive decline up to a decade, specifically in men. The underlying mechanisms may differ by sex, likely through a metabolic driven pathway in men and a hematologic‐inflammatory pathway in women. Future studies investigating omics markers, such as proteomics and metabolomics, may provide additional insight into sex‐specific pathways.

## Author Contributions

Conceptualization: Q.T., L.F.; Formal analysis: Q.T., A.A.; Data curation: A.A.; Writing – original draft: Q.T., A.A.; Writing – review and editing: Q.T., A.A., L.F.; Supervision: L.F.

## Funding

No funding was provided for this study. This research was supported entirely by the Intramural Research Program of the National Institutes of Health (NIH). The contributions of the NIH authors are considered Works of the United States Government. The findings and conclusions presented in this paper are those of the authors and do not necessarily reflect the views of the NIH or the U.S. Department of Health and Human Services.

## Conflicts of Interest

The authors declare no conflicts of interest.

## Supporting information


**Table S1:** Sex differences in a panel of 64 clinical markers.
**Table S2a:** Associations between mitochondrial oxidative capacity and cognitive function in the absence of cognitive impairment and dementia.
**Table S2b:** Associations between mitochondrial oxidative capacity and cognitive function with additional covariate adjustment.
**Table S3a:** Mediation effects of 64 clinical markers on the association between mitochondrial oxidative capacity and cognitive composite score in men.
**Table S3b:** Mediation effects of 64 clinical markers on the association between mitochondrial oxidative capacity and longitudinal changes in cognitive composite score in men.
**Table S3c:** Mediation effects of 64 clinical markers on the association between mitochondrial oxidative capacity and cognitive composite score in women.
**Table S3d:** Mediation effects of 64 clinical markers on the association between mitochondrial oxidative capacity and longitudinal changes in cognitive composite score in women.

## Data Availability

Data analyzed in this study are available upon request by submitting a proposal via the BLSA website portal (https://www.blsa.nih.gov/how‐apply). All requests to access the BLSA datasets are reviewed by the BLSA Data Sharing Proposal Review Committee and are also subject to approval from the NIH Institutional Review Board.
